# Photoluminescence Enhancement in Perovskite Nanocrystals via Compositional, Ligand, and Surface Engineering

**DOI:** 10.3390/ma18174195

**Published:** 2025-09-07

**Authors:** Chae-Mi Lee, Eun-Hoo Jeong, Ho-Seong Kim, Seo-Yeon Choi, Min-Ho Park

**Affiliations:** Department of Materials Science and Engineering, Soongsil University, 369 Sangdo-ro, Dongjak-gu, Seoul 06978, Republic of Korea

**Keywords:** metal halide perovskite nanocrystals, photoluminescent stability, compositional engineering, surface passivation, ligand engineering

## Abstract

Perovskite nanocrystals (PeNCs) have attracted considerable interest as promising materials for next-generation optoelectronic devices owing to their high photoluminescence quantum yield, narrow emission linewidths, simple composition tunability, and solution processability. However, the practical applicability of these NCs is limited by their compositional, thermal, and environmental instabilities, which compromise their long-term operational performance and reliability. Compositional instability arises from ion migration and phase segregation, leading to spectral shifts and unstable emission. Thermal degradation is driven by volatile organic cations and weak surface bonding, while environmental factors such as moisture, oxygen, and ultraviolet irradiation promote defect formation and material degradation. This review describes the recent advances in improving the photoluminescent stability of PeNCs through compositional engineering (A-/B-site substitution), ligand engineering (X-/L-type modulation), and surface passivation strategies. These approaches effectively suppress degradation pathways while maintaining or improving the optical properties of PeNCs. By performing a comparative analysis of these strategies, this review provides guidelines for the rational design of stable and efficient PeNCs for light-emitting applications.

## 1. Introduction

### 1.1. Metal Halide Perovskite Nanocrystals

In recent years, metal halide perovskites (MHPs) have attracted considerable attention as promising materials for next-generation optoelectronic devices owing to their outstanding performance. MHPs, which typically adopt an ABX_3_ crystal structure, exhibit excellent optoelectronic properties including high absorption coefficients, long carrier diffusion lengths, high charge carrier mobility, and low defect densities [1]. These characteristics enable their potential utilization in various applications such as photovoltaics, photodetectors, lasers, and light-emitting diodes (LEDs) [2,3,4,5]. The ease of a low-temperature solution-phase synthesis and tunability of emission wavelengths via composition and size modulation contribute to their high application potential in optoelectronics [6]. In particular, MHP nanocrystals (PeNCs) can achieve high photoluminescence quantum yields (PLQYs) and narrow full widths at half maximum (FWHMs), making them ideal materials for high-color-purity LED applications such as advanced displays and solid-state lighting [7]. Furthermore, compared with conventional emissive organic or inorganic materials that require expensive and high-temperature vacuum deposition processes, PeNCs offer the advantages of a simple low-cost fabrication process while simultaneously enabling high color reproducibility, high external quantum efficiency (EQE), and device operation at low driving voltages [8,9].

MHPs are composed of a combination of A-site monovalent cations like Cs^+^, methylammonium (MA^+^), and formamidinium (FA^+^); B-site divalent metal cations like Pb^2+^ and Sn^2+^; and X-site halide anions like Cl^−^, Br^−^, and I^−^ [10]. In addition, various procedures—such as the hot-injection method [11], ligand-assisted reprecipitation (LARP) [12], wet grinding [13], template methods, microwave-assisted methods, and ultrasonic methods—have been successfully applied to the synthesis of PeNCs [14] (Table 1).

The formation and structural stability of the perovskite phase can be evaluated using Goldschmidt’s tolerance factor (*t* = (*r*_A_ + *r*_X_)/[$\sqrt{2}$(*r*_B_ + *r*_X_)]), which is calculated based on the ionic radii (*r*) of the constituent A, B, and X ions. The crystal structure formed by perovskites varies depending on the value of the tolerance factor. To obtain an ideal cubic perovskite structure, tolerance factor should lie between 0.9 and 1, which corresponds to the most desirable structure for three-dimensional perovskites. However, when the value of tolerance factor deviates from this range, the perovskite structure undergoes distortion, leading to polymorphism, and phase transitions can occur in the crystal. When the tolerance factor is between 0.7 and 0.9, tetragonal and orthorhombic phases coexist and phase transitions occur [21]. By appropriately selecting and combining these ionic components, the structural and optical properties of PeNCs can be precisely engineered for various optoelectronic applications [22].

PeNCs are typically based on 3D perovskite frameworks, which consist of fully interconnected BX_6_ octahedra facilitating charge transport in all directions. This extended lattice connectivity contributes to a high charge carrier mobility and balanced charge injection, both of which are essential for effective radiative carrier recombination in light-emitting devices. Moreover, the 3D framework provides a uniform energy landscape and reduces the probability of trap-assisted recombination, thereby enhancing the PL process. The 6s^2^ electron configuration of the central metal cations (Pb^2+^ or Sn^2+^) further supports rapid radiative recombination by enabling spontaneous charge separation [23]. In addition, surface passivation via ligand coordination or ionic substitution suppresses nonradiative decay pathways, further improving the emission efficiency of PeNCs [24].

Because of these structural advantages, PeNCs typically exhibit narrow emission bandwidths (FWHM < 20 nm). Their emission wavelengths can be tuned from blue to red through compositional or dimensional engineering. These features enable high color purity and precise wavelength control, which are essential characteristics for applications in high-resolution displays and lighting systems with high color rendering indices. The synergy between their structural and electronic properties leads to highly efficient light emission, making PeNCs strong candidates for next-generation optoelectronic materials. Furthermore, the flexible lattice framework allows versatile ionic substitution, enabling fine control over their structural stability, bandgap energy, and emission wavelength [25].

### 1.2. Instability Factors and Their Solutions for PL Enhancements of PeNCs

PeNCs exhibit remarkable PL properties, such as high PLQY values, narrow emission linewidths, and tunable emission wavelengths through compositional control. These advantages render PeNCs highly attractive for a wide range of optoelectronic applications, particularly light-emitting devices. However, the practical utilization of PeNCs remains significantly hindered by their intrinsic limitations in maintaining structural and optical stabilities under real-world operating conditions. PeNC instabilities can be broadly categorized into three major types: compositional, thermal, and environmental instabilities. The degradation of these materials is often triggered by external stimuli such as light, heat, and moisture, and typically results in a reduced emission efficiency, spectral shifts, stronger nonradiative recombination, and shortened device lifetime. Without proper stabilization strategies, these vulnerabilities prevent a long-term and reliable operation of PeNC-based devices in commercial settings [26].

**Figure 1:**
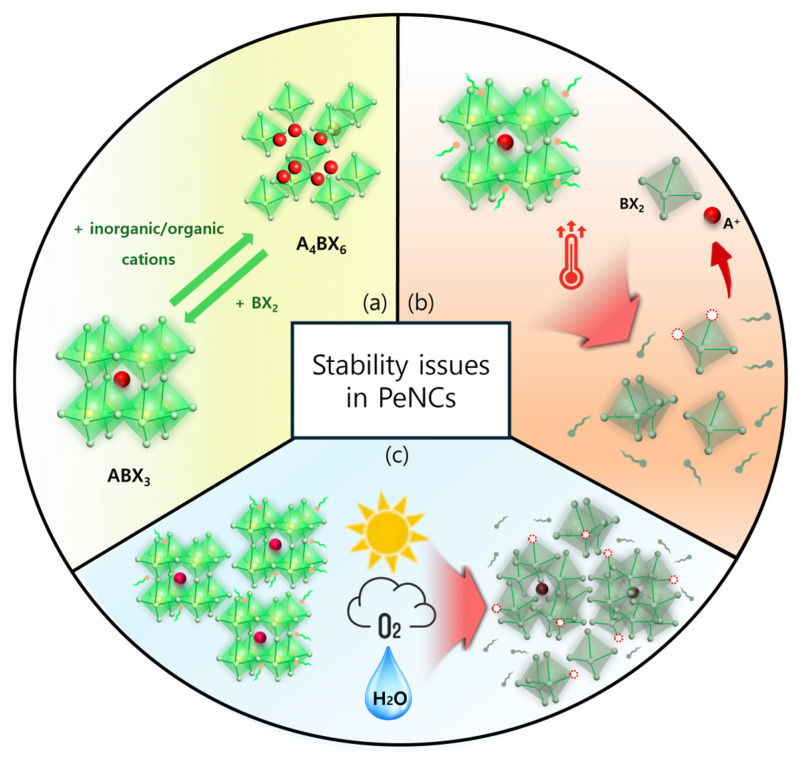
Schematic of the main stability problems of MHP NCs, including (**a**) structural instability, (**b**) thermal degradation, and (**c**) environmental factors.

First, the compositional instability of PeNCs is primarily driven by halide ion migration, which is facilitated by the low activation energy and soft ionic nature of the MHP lattice (Figure 1a). In mixed-halide systems (containing Cl^−^, Br^−^, and/or I^−^ ions), external stimuli such as photoexcitation or electrical bias can trigger ion redistribution, leading to phase segregation, local compositional inhomogeneity, and emission peak instability [27]. These effects are particularly pronounced in nanocrystalline systems with high surface-to-volume ratios and abundant grain boundaries that enhance ion mobility [28,29]. Ion migration causes not only causes dynamic compositional fluctuations but also the accumulation of lattice defects over time. This enhances nonradiative recombination, reduces PLQY, and introduces spatially non-uniform bandgap profiles within bulk films or PeNCs. Such an energetic disorder broadens the emission spectrum and deteriorates device stability and reproducibility. Therefore, the ion migration-induced compositional instability remains a key bottleneck for achieving long-term performance and spectral stability in PeNC-based optoelectronic devices [30].

Second, the thermal instability of PeNCs originates from their susceptibility to structural degradation at elevated temperatures (Figure 1b). MHPs containing A-site cations (such as MA^+^ and FA^+^) decompose at relatively low temperatures (60–100 °C), releasing volatile species such as hydrogen iodide (HI) and methylamine (CH_3_NH_2_), which disrupt the crystal lattice and accelerate material degradation [31]. In PeNCs, the high surface-to-volume ratio and weak ligand binding further exacerbate thermal desorption, leading to surface defect formation, stronger nonradiative recombination, and PL quenching [32]. Moreover, thermal stress can promote particle fusion or localized recrystallization within films, resulting in emission broadening and spatial non-uniformity. These effects collectively degrade the color purity, device efficiency, and operational stability, highlighting the importance of improving the thermal robustness of PeNC-based optoelectronic devices.

Third, environmental instability arises from the high sensitivity of PeNCs to external factors like moisture, oxygen, and ultraviolet (UV) irradiation (Figure 1c). Moisture can penetrate the crystal lattice and induce hydrolysis or ion exchange reactions, leading to the irreversible degradation into lead halide salts (e.g., PbI_2_) and loss of structural integrity. Oxygen exposure under illumination can generate reactive superoxide species (O_2_^−^), which attack organic cations and the halide framework, promoting defect formation and nonradiative recombination. In particular, superoxide species generated under light and oxygen exposure can deprotonate the organic cation (e.g., CH_3_NH_3_^+^), destabilizing the perovskite lattice and leading to decomposition into PbI_2_, I_2_, and H_2_O. The water formed during this process can further accelerate degradation by initiating secondary hydrolysis or hydration reactions, thereby amplifying structural breakdown [33]. UV radiation accelerates these degradation pathways by facilitating halide vacancy formation and photooxidative reactions, particularly on unpassivated surfaces. Collectively, these environmental stressors cause PL quenching, color instability, and reduced device longevity. Therefore, improving the environmental durability of PeNCs requires effective surface passivation and encapsulation strategies [34,35]. In summary, the instability of PeNCs is governed by the complex interplay of compositional, thermal, and environmental factors. Therefore, achieving the long-term durability of PeNC-based optoelectronic devices necessitates the development of integrated strategies that simultaneously overcome all material instability factors.

To address the stability issues, numerous engineering strategies have been proposed to enhance the intrinsic and extrinsic durabilities of PeNCs without compromising their optical performance. Among these strategies, compositional engineering, particularly at A-sites and B-sites, has been extensively explored to improve phase stability, suppress ion migration, and tailor the electronic structure. Substituting volatile organic cations (such as MA^+^ and FA^+^) with more thermally robust inorganic cations (such as Cs^+^) or partially replacing Pb^2+^ with alternative B-site metal cations (such as Mn^2+^) represents an effective method for enhancing structural integrity while mitigating toxicity concerns. In addition, ligand engineering, including ligand exchange as well as X-type and L-type ligand modulation, plays a critical role in stabilizing the surface chemical properties of PeNCs. Carefully designed ligands may not only improve colloidal stability and suppress the formation of surface trap states but also enhance the environmental degradation resistance by passivating undercoordinated Pb^2+^ ions and protecting the PeNC interface. Furthermore, surface passivation approaches, which involve the use of molecular additives, inorganic shells, or multifunctional capping agents, have demonstrated high efficiency in suppressing nonradiative recombination, improving PLQY, and extending operational lifetimes [36].

This paper systematically reviews the recent advances in these key stabilization strategies with a particular focus on how each approach mitigates specific degradation pathways and contributes to the overall enhancement of photophysical properties. Through this comprehensive overview, we aim to provide insights into the design of robust high-performance PeNCs for next-generation optoelectronic devices.

## 2. Engineering Strategies for Enhancing the PL Properties of PeNCs

The PL efficiency of PeNCs is highly sensitive to subtle changes in their structural and chemical environments. While intrinsic properties, such as a high exciton binding energy and defect-tolerant electronic structure, provide favorable material characteristics, the device performance is often compromised by nonradiative pathways arising from ion migration, surface defects, thermal stress, and environmental exposure. To overcome these limitations, extensive research efforts have been devoted to optimizing PeNCs through various engineering strategies. These approaches aim not only to stabilize the crystal lattice and surface chemistry but also to suppress defect-assisted recombination and maintain spectral stability under device operating conditions. The following sections discuss three key aspects that have shown significant promise for enhancing both the PL properties and operational reliability of PeNCs: compositional engineering, surface passivation, and ligand engineering.

### 2.1. Compositional Engineering

PeNCs generally possess an ABX_3_ crystal structure, which is closely related to the Goldschmidt’s tolerance factor depending on the ionic radii of the constituent ions. The tolerance factor is a classical metric that gauges how well the A-site cation geometrically fits into the cavity of a cubic BX_3_ framework. Goldschmidt’s tolerance factor formulated the expression by treating all ions as hard spheres. However, in hybrid systems—e.g., those with multiple A-site cations on the A sublattice and/or mixed metal ions on the B sublattice—the non-sphericity of ions limits the conventional definition of the effective ion radii r_A_ and r_B_. To address this, Becker et al. proposed effective ion radii for 18 molecular A-site cations using gas-phase, energy-minimized electron densities and an isocharge radius referenced to [NH_4_]^+^ [37], and Palgrave et al. re-evaluated divalent B-site radii for halide bonding environments and introduced the octahedral factor μ as an additional parameter to assess B–X size mismatch [38]. In summary, calculating tolerance factor with a chemically consistent set of A/B-site effective radii overcomes the limitations of the hard-sphere approximation and provides a concise indicator of phase formation and distortion in hybrid perovskites. At present, the combination of Becker’s values for the A-site and Palgrave’s halide-adjusted B-site radii appears most appropriate [39]. Through these studies, the tolerance factor(t) range required to obtain an ideal cubic perovskite structure has been established as 0.9 < t < 1 [37,38]. Deviations from this range can induce phase transitions or structural distortions. To regulate this structural sensitivity, compositional engineering has emerged as a key strategy for enhancing the stability of PeNCs. This approach involves the substitution or doping A-site, B-site, and X-site ions with alternative ions. Typically, inorganic or organic cations such as Cs^+^, MA^+^, and FA^+^ ions are doped at the A-sites, and transition metal ions such as Sn^2+^, Mn^2+^, and Zn^2+^ ions are doped at the B-sites.

Compositional changes introduce internal lattice strain and local distortions whenever the dopant radius mismatches the host site, thereby modifying the electronic structure and shifting the bandgap/PL peak. Typically, incorporation of a larger ion expands the lattice, lengthens the lead–halide bonds, and reduces the bandgap, producing a red- shifted PL. In MHPs, studies report that larger cations correlate with smaller bandgaps [40,41]. Conversely, introducing a smaller ion contracts the lattice, shortens the Pb–X bond length, and increases Pb–X orbital overlap, thereby widening the bandgap and inducing a blue shift in PL emission [42].

Beyond ionic-size effects, noncovalent interactions can also play a decisive role in governing structure and bandgap. Density functional theory (DFT) analyses of the Cs, MA, FA cations indicate that FA exhibits a statistically higher tendency for forming hydrogen bonds, which stabilizes a cubic framework. In this geometry, the increased ionic character of Pb–I bonds enhances the Pb contribution near the conduction-band edge, thereby amplifying spin–orbit coupling (SOC) and further reducing the bandgap. These results underscore that electronic structure and emission wavelength are jointly determined by the interplay among cation size, hydrogen bonding, octahedral tilting, and SOC [43].

**Figure 2:**
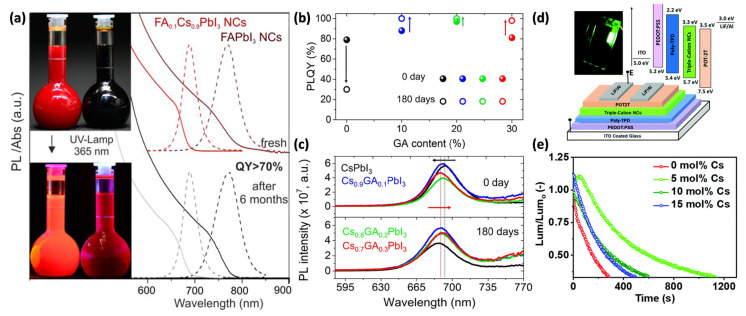
(**a**) Optical absorption and photoluminescence (PL) spectra of FAPbI_3_ and FA_0.1_Cs_0.9_PbI_3_ NCs recorded before and after six months of storage. The insets contain photographs of the toluene PeNCs colloids (left: FAPbI_3_, right: FA_0.1_Cs_0.9_PbI_3_)—top: in daylight; bottom: under 365 nm UV irradiation. Reprinted with permission from Ref. [45]. © 2017 American Chemical Society. (**b**) Photoluminescence quantum yield (PLQY) and (**c**) PL spectra of Cs_1−x_GA_x_PbI_3_ PeNCs with different GA contents recorded at 0 and 180 d. Reprinted with permission from Ref. [46]. © 2023 The Authors. Published by the American Chemical Society. Panels (**d**,**e**) display the device structure and stability characteristics of the Cs_x_(MA_0.17_FA_0.83_)_1−x_PbBr_3_-based light-emitting diodes (LEDs) (x = 0–0.15). (**d**) Schematic diagram and energy levels along with a photograph of the 5 mol% Cs device operated at 3.00 V. (**e**) Normalized luminance (Lumin/Lumin_0_) over time plotted under constant current driving conditions. Reprinted with permission from Ref. [47]. © The Royal Society of Chemistry 2020.

Overall, compositional engineering provides an effective route to simultaneously optimize the structural robustness and optoelectronic performance of PeNCs (e.g., PLQY, spectral positioning, bandgap matching) by judicious selection of the substitution site (A, B, X), balancing dopant radius and chemical interactions, and incorporating crystallographic considerations. In the ABX_3_ structure, the A-site cation is located in the [BX_6_]^4−^ octahedral framework and plays a crucial role in determining the overall shape and stability of the crystal structure. When the ionic radius of a dopant at the A site is too large or too small, it can induce distortions with respect to the B–X lattice, resulting in tilted B–X–B bond angles and local lattice strain [44]. Hence, only ions with suitable radii can be stably incorporated, which limits the variety of suitable dopant ions. Commonly used A-site dopants include organic cations such as MA^+^, FA^+^, and GA^+^, as well as the inorganic cation Cs^+^. Protesescu et al. synthesized monodisperse, cubic FAPbI_3_ and FA_0.1_Cs_0.9_PbI_3_ NCs using formamidinium acetate (FAOAc), cesium carbonate (Cs_2_CO_3_), and lead(II) iodide (PbI_2_) as precursors, where FAPbI_3_ showed a PL emission peak at approximately 770–780 nm and FA_0.1_Cs_0.9_PbI_3_ at 685 nm. Both samples maintained PLQYs of ≥70%, exhibited excellent structural and chemical stability, and retained stable emission for several months at room temperature (Figure 2a) [45]. Furthermore, Serafini et al. reported that, by using mixed guanidinium acetate (GAOAc), Cs_2_CO_3_, and PbI_2_ precursors, doping CsPbI_3_ NCs with 10 mol% GA^+^ significantly enhanced the PLQY and PL intensity, with these properties remaining stable for 180 days under refrigerated storage (Figure 2b,c) [46].

A double-cation strategy, involving a mixture of two cations, is typically employed in A-site engineering. However, in the case of MA/FA double-cation compositions, intrinsic structural and thermal instabilities make the fabrication process highly sensitive to processing conditions. This sensitivity often leads to the formation of detrimental impurities within the film and reduces crystallinity, resulting in significant performance variability. To address these issues, recent studies have focused on triple-cation compositions (Cs/MA/FA) that incorporate three different A-site cations. In particular, the inclusion of Cs^+^ ions into the triple-cation strategy plays a crucial role in enhancing both the process stability and structural robustness. The incorporation of a small amount of the inorganic cation Cs^+^ helps optimize the Goldschmidt tolerance factor, thereby improving the structural stability of the perovskite crystal lattice. Additionally, it promotes monolithic grain formation, which increases the phase purity [48]. Vashishtha et al. synthesized Cs_x_(FA_0.83_MA_0.17_)_1−x_PbBr_3_ NCs via LARP method, using cesium bromide (CsBr), formamidinium bromide (FABr), methylammonium bromide (MABr), and lead(II) bromide (PbBr_2_) as precursors. The resulting triple-cation NCs PLQY of up to ~93% and were employed as the EML to fabricate green PeLEDs (Figure 2d). The devices achieved a maximum EQE of 7.4% (30 cd/A) and current efficiency. From device lifetime measurement with the initial luminance (Lumin_0_) values for devices of 77.4, 97.7, 107.4, and 110.1 cd/m^2^(0, 5, 10, and 15 mol% Cs), the 5 mol% Cs device exhibited the highest operational stability (Figure 2e. These findings indicate that dopant engineering aimed at improving PL characteristics is a practical and effective strategy that can positively translate into enhanced EL performance.

However, organic cations are thermally unstable and prone to degradation at high temperatures. To overcome this limitation, using thermally stable inorganic cations as dopants has been investigated. Ko et al. synthesized Cs_1−x_Rb_x_PbBr_3_ NCs incorporating up to 43% rubidium ions (Rb^+^), using a mixed A-site cation precursor solution of rubidium carbonate (Rb_2_CO_3_) and Cs_2_CO_3_, demonstrating bandgap widening and emission wavelength tuning (from 515 to 494 nm) due to a lattice distortion [49]. Although A-site doping is an effective method for enhancing structural stability and optical properties, the selection of dopant ions remains fundamentally limited.

B-site doping is another prominent strategy for improving the structural stability of PeNCs, typically achieved by substituting Pb^2+^ ions with transition metal ions. In addition to lattice stabilization, B-site doping modifies the bandgap and enhances luminescence by mitigating surface defects, such as halide vacancies [50]. Although Pb^2+^ doping is favored because of the high charge mobility and crystallinity, the toxicity of Pb^2+^ ions restricts their practical applications. Therefore, B-site doping has also been pursued to partially replace Pb^2+^ ions, albeit with challenges due to the higher formation energy and risk of degrading the optoelectronic properties of LHPs after completely substituting Pb^2+^ species [51].

**Figure 3:**
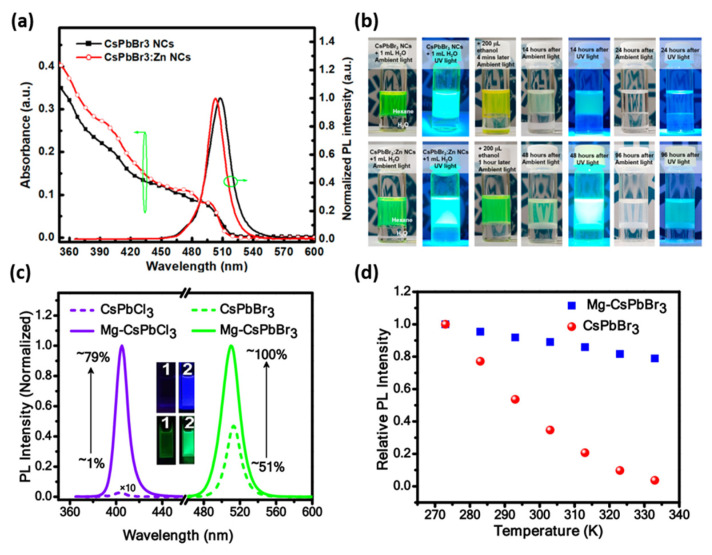
(**a**) Absorbance and PL profiles of the CsPbBr_3_ and Zn-doped CsPbBr_3_ NCs dispersed in hexane. (**b**) Top: CsPbBr_3_; bottom: CsPbBr_3_:Zn. The leftmost two panels show NCs dispersions in hexane. Panels 3–7 present time-lapse photoluminescence images after injection of 200 μL ethanol at the indicated time points. Reprinted with permission from Ref. [52]. © 2022 The Authors. Licensee MDPI, Basel, Switzerland. (**c**) PL intensities and PLQYs of pristine and Mg-doped CsPbCl_3_ and CsPbBr_3_ NCs. (**d**) Temperature-dependent PL intensity of CsPbBr_3_ NCs plotted with and without Mg doping. Reprinted with permission from Ref. [53]. © 2020 American Chemical Society.

B-site dopants typically include divalent ions such as Mn^2+^ and Zn^2+^, whose charges are comparable to that of Pb^2+^. Doping with a smaller ion induces lattice contraction, shortens the Pb–X bond length, and enhances orbital interactions, thereby increasing the bandgap. Zeng et al. reported that Zn^2+^ doping in CsPbBr_3_ NCs, synthesized using Cs_2_CO_3_, PbBr_2_, and zinc(II) bromide (ZnBr_2_) precursors, effectively passivated Pb surface vacancies and improved moisture stability. In Figure 3a, the absorption and normalized PL spectra of Zn-doped CsPbBr_3_ NCs display a narrower FWHM and a more pronounced first excitonic absorption peak, indicating a more uniform size distribution and higher color purity. For the moisture-resistance test, 1 mL of water was added to hexane dispersions of CsPbBr_3_ and CsPbBr_3_:Zn NCs at identical concentrations. Because the large polarity contrast between hexane and water initially suppressed measurable PL decay over several days, hydrolysis was accelerated by adding 200 μL of ethanol. The pristine CsPbBr_3_ NCs dispersion turned yellow within minutes and lost colloidal stability, becoming transparent within one day, whereas the CsPbBr_3_:Zn NCs dispersion retained green emission and showed markedly delayed degradation for more than four days (Figure 3b). These results suggest that Zn-induced passivation of surface Pb vacancies blocks water ingress and subsequent decomposition pathways, thereby enhancing the moisture stability of all-inorganic lead halide PeNCs [52].

Das et al. synthesized Mg^2+^-doped CsPbX_3_ (X = Cl or Br) NCs with excellent thermal and optical properties, in which magnesium chloride (MgCl_2_) precursor was added during the synthesis to introduce Mg^2+^ doping. Mg^2+^ doping led to a lattice contraction, suppressed thermally induced carrier trapping, significantly enhanced PLQY, and retained ~85% of the initial emission intensity even at 333 K for CsPbBr_3_ (Figure 3c,d) [53]. Kim et al. demonstrated improved photophysical properties and environmental stability of CsPbBr_3_ NCs doped with Ni^2+^ ions, synthesized using Cs_2_CO_3_, PbBr_2_, and nickel(II) bromide (NiBr_2_) precursors. At the optimal Ni/Pb ratio of 2.5, the PL intensity increased by a factor of 3.8, PL lifetime was extended by 13.7 ns, and quantum yield improved by 26.2% [54]. Chen et al. reported enhanced structural and emission stabilities of CsPbI_3_ NCs doped with Cu^2+^ ions, using copper(II) acetate (Cu(OAc)_2_) as the precursor for Cu^2+^ doping. The optimal doping conditions increased PLQY from 68.6% to 74.2% and extended the lifetime from 28.5 to 48 ns. Moreover, the PL spectrum was maintained over 35 d in air [55]. Trivalent-ion doping also improved the structural and optical properties of MHPs via charge redistribution and defect suppression. Guvenc et al. reported that doping CsPbI_3_ NCs with 10 mol% Gd^3+^ enhanced their phase stability and emission characteristics, using Cs_2_CO_3_, PbI_2_, and gadolinium(III) acetate (Gd(OAc)_3_) as precursors. The PLQY value increased from 70% to 80%, and average PL lifetime increased from 47.4 to 64.4 ns [56].

In conclusion, compositional engineering is an effective strategy for simultaneously improving the structural stability and photophysical properties of PeNCs. Depending on the doping site, it enables control over lattice distortion and defect suppression, thus enhancing the long-term stability and device performance.

### 2.2. Surface Passivation

PeNCs possess large surface areas, making them more susceptible to defect formation than bulk single crystals or polycrystals. To elucidate the origin of defects in PeNCs, Ten Brinck et al. investigated a possible formation mechanism of trap states in well-defined nonperiodic CsPbBr_3_ NC models with sizes of approximately 3 nm. Using density functional theory (DFT), they calculated the formation energies of interstitial, vacancy, and anti-site defects in various NC regions (center, surface center, and surface edge). Their results confirmed that the most stable defects were formed on the surface [57]. Owing to the ionic nature of PeNC surfaces, degradation occurs readily, increasing the instability and deterioration of optical properties. Therefore, surface defect passivation strategies are essential for enhancing the stability of PeNCs. To determine a fundamental relationship between the surface chemistry and optoelectronic properties of PeNCs, Li et al. suggested that the self-passivation effect of halide atoms correlated with a high PLQY. Abundant halide atoms on the surface region passivate uncoordinated Pb^2+^ cations and inhibit the trapping of excited electrons at surface defects, facilitating efficient radiative recombination. Furthermore, under halide-rich synthesis conditions (when the surface is enriched with halide ions), excess halide ions fill surface halide vacancies, reducing nonradiative recombination pathways and maximizing the PLQY value. Consequently, high PLQYs of approximately 80%, 95%, and 70% were achieved for red, green, and blue emissions, respectively [58].

**Figure 4:**
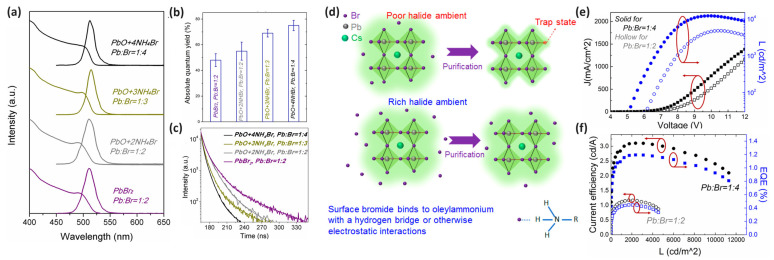
(**a**) PL and UV spectra of NCs synthesized using different precursors at various halide contents. (**b**) Average QY values of NCs. (**c**) Time-resolved PL (TRPL) decay and fitting curves of the purified CsPbBr_3_ NCs. (**d**) Schematics describing the halide-poor and halide-rich conditions for the synthesis of NCs. (**e**) Performance characteristics of devices with different NCs, including the current density (CD) and luminance (L) plotted as functions of the driving voltage. (**f**) Current efficiency (CE) and external quantum efficiency (EQE) plotted as functions of luminance. Reprinted with permission from Ref. [59]. © 2017 American Chemical Society.

Liu et al. demonstrated that halide-rich environments significantly enhanced the optoelectronic properties and stability of CsPbBr_3_ PeNCs. By modulating the Pb:Br molar ratio from the conventional 1:2 to higher bromine concentrations of 1:3 and 1:4, substantial improvements in the emission properties were observed while maintaining consistent emission wavelengths. Spectroscopic analysis data revealed that increasing the Br concentration narrowed FWHM from 25 to 19 nm while maintaining the emission wavelength at 512–514 nm (Figure 4a) and dramatically increased PLQY from 48% to 75% (Figure 4b) [59]. Time-resolved PL (TRPL) measurements confirmed the reduced number of electron trap states in halide-rich environments, indicating effective defect passivation (Figure 4c). A long TRPL lifetime generally arises when electrons or holes are trapped in defect states, where they remain for extended periods without undergoing non-radiative recombination. Therefore, a shortening of the long-lifetime component in TRPL signals indicates that fewer carriers are trapped, allowing recombination processes to proceed more efficiently and rapidly. This serves as a clear indication of successful defect passivation. Figure 4d illustrates the difference between PeNCs synthesized in halide-poor and halide-rich environments, showing how excess halides maintain surface stability even after purification processes that typically remove protective ligands [59]. The practical significance of this approach was verified through LED fabrication, in which devices incorporating halide-rich PeNCs demonstrated remarkable performance enhancements. As shown in Figure 4e,f, the maximum luminance, current efficiency, and EQE of PeLEDs increased by more than two times as compared with that of conventional PeNCs, and the turn-on voltage decreased from 5.8 to 4.6 V. This halide-rich synthesis strategy represents an efficient approach to addressing the persistent challenges of defect states in PeNCs. By simply adjusting the precursor stoichiometry, significant improvements in both the PL properties and device performance can be achieved without introducing additional processing steps or foreign passivation agents [59].

Surface defect passivation in PeNCs has been achieved through element compensation strategies that aim to eliminate surface vacancies by providing necessary elements [60]. Metal halide precursors have been effectively employed to provide metals and halide ions for surface passivation. Several studies have demonstrated that the surface passivation of PeNCs enhances their optical and electrical properties [61,62].

**Figure 5:**
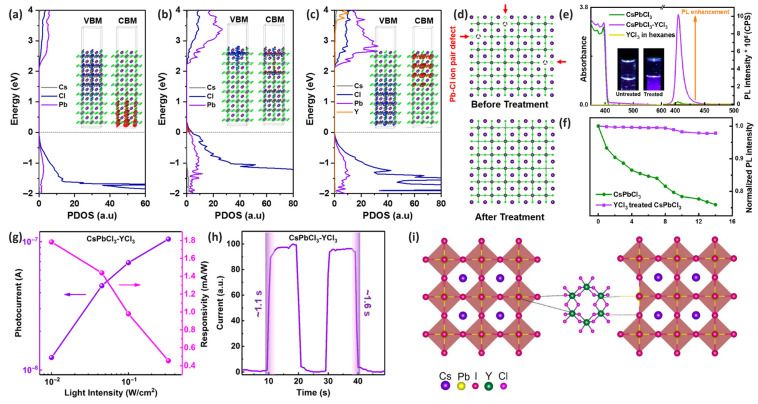
Projected densities of states and electronic charge densities for the valence band maximum (VBM) and conduction band maximum (CBM) determined for (**a**) an ideal CsPbCl_3_ slab, (**b**) a slab with a removed Pb–Cl ion pair, and (**c**) a slab with an added Y–Cl ion pair. (**d**) Schematic representation of the crystal structure of CsPbCl_3_ NCs and mechanism proposed for the YCl_3_ surface treatment. (**e**) Steady-state optical absorption and PL spectra recorded for the as-synthesized CsPbCl_3_ NCs dispersed in hexanes before and after the YCl_3_ surface passivation. The insets contain photographs of the untreated and YCl_3_-treated NCs under 365 nm UV light. (**f**) Relative stability test represented by the normalized PL intensity plotted as a function of time (days) for pristine and YCl_3_-passivated NCs after the exposure to ambient conditions. The PL spectrum was obtained upon a 365-nm excitation. (**g**) Photocurrent (left) and responsivity (right) at −10 V plotted as functions of the UV power density. (**h**) Temporal photoresponse of CsPbCl_3_–YCl_3_ under alternating dark and UV light. Reprinted with permission from Ref. [60]. © 2018 American Chemical Society. (**i**) Intentional doping of YCl_3_ in the CsPbI_3_ NC precursor solution. The presence of surface chloride species initiates a surface energy imbalance of the unit cell, leading to the anisotropic growth of CsPbI_3_ NCs. Reprinted with permission from Ref. [63]. © 2023 Saleem et al., Licensee MDPI, Basel, Switzerland.

Ahmed et al. employed YCl_3_, a trivalent metal chloride, as a post-synthesis treatment for the dual surface passivation of CsPbCl_3_ NCs. This approach effectively passivated surface defects while maintaining the NC size and crystal structure. The dual passivation mechanism involves the formation of Y–Cl ion pairs that passivate Pb–Cl ion pair defects on the surface. DFT calculation results revealed that on the ideal CsPbCl_3_ NC surface, the electron and hole wave functions were well separated (Figure 5a), whereas Pb–Cl ion pair defects created trap states above the conduction band with holes localized on the surface (Figure 5b). When Y–Cl ion pairs fill these defect sites, they enhance the density of states in the conduction band region without creating midgap states (Figure 5c) [60].

Spectroscopic measurements demonstrated that YCl_3_ treatment induced minimal changes to the absorption and emission properties of CsPbCl_3_ NCs with emission peaks showing less than 1-nm red shifts while maintaining a narrow FWHM of 11 nm. Significantly, the PL intensity increased by approximately 60 times after the YCl_3_ treatment (Figure 5e) [60]. This improvement provides clear experimental evidence for the surface defect passivation of PeNCs [64]. An investigation of the YCl_3_ surface treatment effects on CsPbCl_3_ NCs revealed significant differences in the photostability performance between the treated and untreated samples. While the untreated CsPbCl_3_ NCs exhibited a 30% decrease in the PL intensity after two weeks, the YCl_3_-treated NCs maintained nearly 100% of their initial PL intensity throughout the 14-d testing period (Figure 5f) [60]. These results demonstrate that YCl_3_ treatment effectively eliminated surface defects while enhancing the long-term durability of the optical properties of the nanomaterials [65]. The photoelectric performance of the YCl_3_-passivated CsPbCl_3_ NCs was evaluated using flexible device arrays on polytetrafluoroethylene membranes. Devices fabricated with the YCl_3_-treated NCs showed enhanced photoconductivity at low power densities and a 40% reduction in the temporal photoresponse rise time (Figure 5g,h). Other trivalent metal chlorides (InCl_3_, LaCl_3_, GdCl_3_, and YbCl_3_), which can be attributed to the high affinity of trivalent ions, exhibited similar improvements [60].

Using a different approach, Saleem et al. incorporated YCl_3_ into CsPbI_3_ NCs during synthesis, exploiting the binding energy differences between the chloride and iodide ions on the CsPbI_3_ NC surface and lattice to induce anisotropic growth (Figure 5i). This approach resulted in the formation of one-dimensional CsPbI_3_ nanorods with lengths of 18.5 nm and diameters of 8.2 nm rather than conventional cubic-structured NCs. These YCl_3_-treated nanorods exhibited a PLQY increase from 51% to 70% and superior photostability, demonstrating excellent durability with only a 28% decrease in PLQY from 70% to 50% after 45 d of storage at room temperature, whereas the PLQY of pristine CsPbI_3_ NCs approached zero over the same period. When the YCl_3_-treated CsPbI_3_ nanorods were applied to the emitting layers of PeLEDs, the passivated CsPbI_3_ nanorods achieved an EQE of 3.16%, representing a 1.86-fold improvement over the untreated CsPbI_3_ NC LED. These studies highlight the versatility and effectiveness of trivalent metal halides, particularly YCl_3_, for passivating surface defects of PeNCs through dual-ion compensation mechanisms, significantly enhancing their optical properties and device performance [63].

Generally, PeNCs are capped with long-chain ligands such as oleic acid (OA) and oleylamine (OLA) [66]. OLA is primarily utilized to control the growth kinetics of PeNCs, whereas OA serves as a stabilizing agent. These surface ligands play crucial roles in determining the colloidal stability, optical properties, and overall performance of PeNCs for various applications [67].

**Figure 6:**
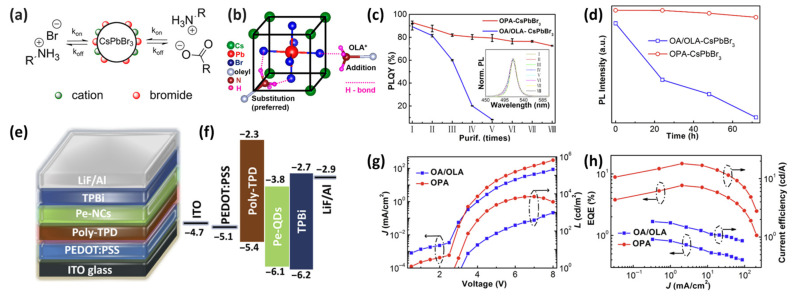
(**a**) Schematic illustrating possible binding of oleylamine (OLA) to CsPbBr_3_ by forming hydrogen bonds with Br^−^ ions. Reprinted with permission from Ref. [68]. © 2016 American Chemical Society. (**b**) Schematic representation of the dynamic surface stabilization by oleylammonium bromide. Reprinted with permission from Ref. [69]. © 2017 American Chemical Society. (**c**) PLQY of OPA-CsPbBr_3_ and OA/OLA-CsPbBr_3_ NC solution in hexane with one to eight purification cycles. Inset shows the PL spectra of OPA-CsPbBr_3_ NC solution with different purification cycles. (**d**) Time dependence of PL intensity of OPA-CsPbBr_3_ and OA/OLA-CsPbBr_3_ NC films stored in ambient atmosphere (humidity ∼50%) (**e**) Schematic device structure. (**f**) Energy level diagram of the devices. (**g**) J–V–L and (**h**) EQE–J–E curves of the devices based on different ligands [67]. © 2018 American Chemical Society.

To elucidate the passivation mechanism of organic ligands on CsPbBr_3_ NC surfaces, Ravi et al. examined the unique interactions between the organic capping ligands and inorganic cores using X-ray photoelectron spectroscopy (XPS), nuclear magnetic resonance (NMR), and DFT calculations. Based on the obtained results, two passivation mechanisms are proposed. First, the addition (or adsorption) of OLA on the PeNC surface facilitates the formation of hydrogen bonds between OLA and Br^−^ species (Figure 6a) [68]. Second, OLA substitutes Cs^+^ ions on the PeNC surface. Notably, the latter mechanism is more favorable owing to the formation of additional H⋯Br hydrogen bonds. Furthermore, both mechanisms involve distinct surface reconstruction types, which significantly influence the results of the surface passivation process (Figure 6b) [69].

The stability of CsPbBr_3_ NCs is significantly limited by the weak interactions between OA/OLA ligands and the CsPbBr_3_ NC surface, drastically deteriorating the PLQY and dispersibility in solvents. Ligands exhibiting strong interactions with CsPbBr_3_ NCs have been extensively investigated to enhance the stability of CsPbBr_3_ NCs. Tan et al. demonstrated that octylphosphonic acid (OPA) could be employed as a replacement for conventional OA/OLA ligands, whereby the stability of CsPbX_3_ NCs was dramatically improved. The two chelating binding sites of OPA strongly coordinate Pb ions in CsPbX_3_ NCs, resulting in robust surface passivation. Consequently, the OPA-capped CsPbBr_3_ NCs exhibit a high PLQY (~90%) and narrow FWHM (~19 nm). Notably, a high PLQY (≥80%) was maintained in the OPA-capped CsPbX_3_ NCs (Figure 6c), and colloidal stability was preserved even after multiple purification processes (Figure 6d). In contrast, under the same conditions, the PLQY of the OA/OLA-capped CsPbBr_3_ NCs decreased sharply to approximately 20% (Figure 6c) [67]. In addition to OPA, other ligands such as trioctyl phosphine oxide (TOPO) [70] and bis-(2,2,4-trimethylpentyl) phosphinic acid [71] effectively passivate and stabilize PeNCs because of their strong interactions with the NC surface. 

Green PeLEDs based on the OPA-capped CsPbBr_3_ NCs (Figure 6e,f) exhibit significantly higher current efficiency (18.13 cd A^−1^) and EQE (6.5%) as compared with those of conventional OA/OLA-capped CsPbBr_3_ NC LEDs (Figure 6g,h) [67]. The superior performance of the OPA-capped CsPbBr_3_ NC LEDs was attributed to the higher PLQY of PeNCs and formation of a uniform high-quality thin film. 

Several deficiencies were observed in the OA/OLA-capped CsPbBr_3_ NC LEDs, including an elevated leakage current, poor film morphology, and increased number of surface trap states resulting from the ligand desorption [67]. With respect to the long-term operational stability, the EQE of the OPA-capped CsPbBr_3_ NC LEDs retained more than 50% of their initial EQE after being subjected to constant current conditions for 30 min. Thus, these devices were more stable than the OA/OLA-capped CsPbBr_3_ NC LEDs, which maintained only 20% of their initial EQE [67].

Tang et al. reported a synergistic surface passivation approach using OPA and (3-aminopropyl) triethoxysilane (APS) to improve the long-term stability of CsPbI_3_ NCs. OPA exhibited a high binding affinity for passivating surface defects, while APS not only passivated surface defects but also offered active sites for the formation of a SiO_2_ layer on the PeNCs surface, which further mitigated ligand detachment and effectively blocked external degrading factors (such as moisture and oxygen) from accessing PeNCs [72]. The stability of the OPA/APS-capped CsPbI_3_ NCs was synergistically enhanced as compared with that of conventional OA/OLA-capped PeNCs with a significant improvement in their PL properties. When OA and OLA were replaced with OPA and APS in CsPbI_3_ NCs, the PLQY value significantly increased from 30.1% to 73.3%, demonstrating excellent photophysical properties and superior defect passivation [72]. Moreover, while complete PL quenching occurred within 7 d for the OA/OLA-capped CsPbI_3_, the OPA/APS-capped CsPbI_3_ maintained 80.5% of its initial PL intensity even after 120 d of ambient storage, revealing outstanding long-term PL stability. Utilizing these high-performance OPA/APS-capped CsPbI_3_ PeNCs as color-converting materials, white LEDs with color coordinates of (0.33, 0.32) and an impressive color gamut reaching 126% of the National Television Standard Committee color space were fabricated [72].

### 2.3. Ligand Engineering

PeNCs are typically synthesized via colloidal routes and stabilized by organic ligands bearing surface-binding functional groups. These ligands not only maintain colloidal stability, but also play critical roles in the synthesis and application processes by influencing crystallinity, optical properties, charge transport, and structural stability [73]. OLA and OA are among the most widely used ligands, which help suppress nonradiative recombination pathways and enhance PL properties. However, these ligands have several limitations.

First, a reversible proton transfer reaction occurs between OA and OLA (OA^−^ + OLAH^+^ ⇌ OLA + OA; OLAH^+^ + X^−^ ⇌ OLA + HX), leading to continuous fluctuations in the surface-bound ligand species [74]. This dynamic acid–base equilibrium prevents stable surface passivation. Second, owing to their dynamic equilibrium, OA and OLA repeatedly adsorb and desorb from the NC surface, which induces the formation of surface defects and disrupts the uniformity of crystal growth. Furthermore, these ligands possess inherently weak binding affinities for the ionic surface of PeNCs, making them prone to detachment and thus ineffective in ensuring long-term stability enhancement. As a result, the OA/OLA ligand pair introduces significant uncertainty in controlling crystal growth and achieving long-term colloidal and structural stability. This increases the nonradiative recombination rate and poses fundamental limitations for improving the photoluminescence efficiency [68]. 

Long-chain alkyl ligands also strongly influence charge transport because they behave as electrical insulators and can impede the charge current flow in MHP thin films. To address this limitation, significant efforts have been made to introduce short-chain ligands or optimize the ligand density [75]. Accordingly, to overcome the kinetic lability, long-term stability issues, and charge-transport limitations associated with the conventional OA/OLA ligand pair, a broad set of ligand-engineering strategies has been widely employed. Experimentally, these include (i) post-synthetic ligand treatments, in which additional ligands are introduced after NC formation, and (ii) ligand-exchange protocols that replace the native ligands with more suitable ones. However, such approaches inevitably increase the number of synthetic steps and complicate processing. To mitigate these drawbacks, one-pot syntheses that introduce functional ligand precursors during PeNCs growth are being actively explored, offering a pathway to simplify processing while simultaneously enhancing surface passivation and device characteristics [76,77].

Ligand engineering is a key strategy for improving the structural, photophysical, and optoelectronic stability of PeNCs. Ligands can directly influence the crystal growth dynamics, defect passivation, charge transport, and phase retention. Therefore, the selection and design of ligands are fundamental requirements for the development of stable and high-performance PeNCs.

#### 2.3.1. L-Type Ligands

**Figure 7:**
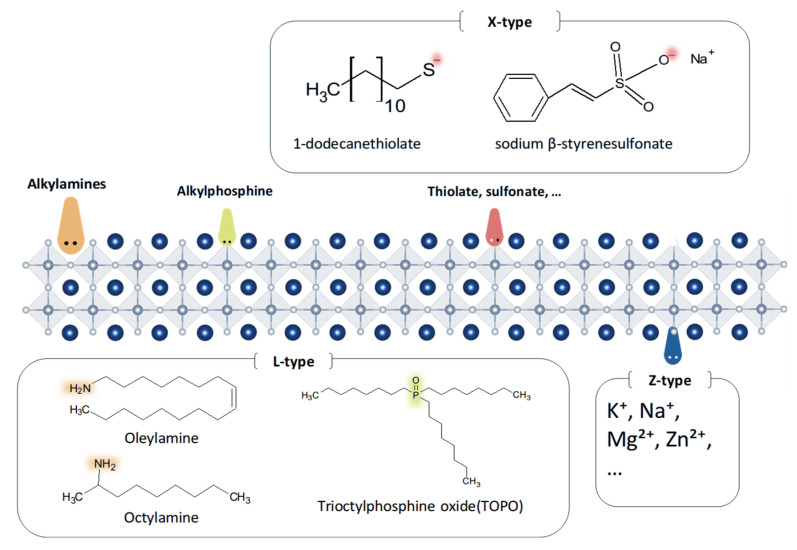
Schematic illustration of the L-type, X-type, and Z-type ligands.

Perovskite ligands are broadly classified into L-type, X-type, and Z-type ligands depending on their coordination interactions with surface species. This classification is based on the mode of the electron exchange between the ligands and unstable surface ions, particularly metal cations (such as Pb^2+^) and halide anions (such as Br^−^) exposed on PeNC surfaces. L-type ligands are neutral Lewis bases that donate lone pairs of electrons to coordinate with metal centers. In contrast, Z-type ligands are neutral Lewis acids that accept lone pairs of electrons from metals. X-type ligands are anionic species that donate one electron to form covalent bonds with a metal center (Figure 7) [78].

Representative L-type ligands include alkylamines (such as OLA and octylamine) and alkylphosphine-based ligands (such as trioctylphosphine oxide and TOPO). L-type ligands form coordination bonds with metal cations via lone-pair electrons in their molecular structure and selectively bind to undercoordinated Pb^2+^ ions on the surface of PeNCs, thereby effectively passivating surface defects. Because of their strong electron-donating ability and stable coordination behavior, they are widely employed to improve the optical and structural stabilities of MHPs [79].

Zhao et al. developed a complementary triple-synergy ligand strategy by combining an organic–inorganic hybrid ligand system consisting of a conjugated ligand, 3-phenyl-2-propen-1-amine (PPA); an inductive inorganic ligand, ZnBr_2_; and a long-chain ligand, didodecyldimethylammonium bromide (DDAB). The introduction of conjugated ligands has attracted considerable attention as a promising alternative as they can simultaneously passivate surface defects and enhance charge transport between PeNCs owing to the delocalized electrons in their conjugated π-orbitals. However, conjugated ligands typically exhibit insufficient passivation and a tendency to detach easily, which destabilize the Pb–Br octahedral framework and increase nonradiative recombination pathways, ultimately reducing the PLQY value [80].

**Figure 8:**
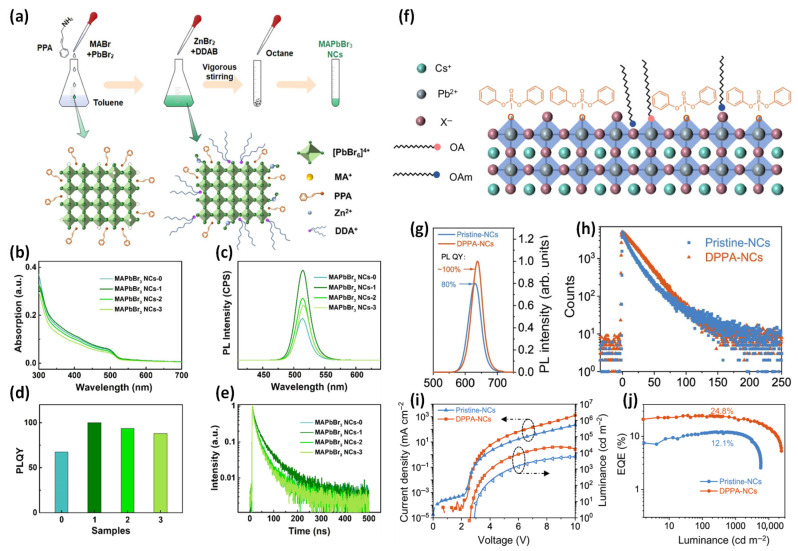
(**a**) Schematic illustration of the preparation of MAPbBr_3_ NCs. (**b**) UV–vis absorbance, (**c**) PL, (**d**) PLQY, and (**e**) TRPL spectra of MAPbBr_3_ NCs with different synergistic ligand concentrations. Reprinted with permission from Ref. [80]. © 2022 American Chemical Society. (**f**) Schematic illustration of the diphenylphosphoryl azide (DPPA) NC surface. (**g**) PL spectra and (**h**) TRPL decay curves of NCs in solution. (**i**) Current density–voltage–luminance and (**j**) EQE−luminance characteristics of the PeLEDs. Reprinted with permission from Ref. [81] © 2025 Springer Nature Limited.

To address this limitation, post-synthetic ligand exchange using polar solvents is commonly employed. However, this approach can damage the ionic crystal structure of PeNCs and compromise their structural stability. In this study, PPA was introduced during the early growth stage of MAPbBr_3_ NCs via a modified LARP method, thereby facilitating nucleation and crystal growth. Subsequently, a ligand solution containing ZnBr_2_ and DDAB dispersed in toluene without polar solvents was injected in situ to further passivate surface defects and tune the ligand environment (Figure 8a). The absolute intensities of the UV–vis absorption and PL spectra varied with the ligand composition. As the ZnBr_2_ concentration increased, the PL intensity initially increased and then gradually decreased (Figure 8b,c; denoted as MAPbBr_3_ NCs-x, where x = 0, 1, 2, and 3 correspond to the ZnBr_2_-to-DDAB of 0:5.75, 1:5.75, 1:2.88, and 1:1.92, respectively). An increase in the ZnBr_2_ content increased the number of short-chain ligands capping the NC surface, which facilitated NC growth and induced a PL peak shift. However, this aggregation deteriorated the PL intensity, PLQY, and TRPL characteristics (Figure 8d).

TRPL measurement results revealed that the introduction of synergistic ligands increased the average lifetime from 31.00 to 41.62 ns, and the radiative recombination ratio improved from 45.80% to 54.42% (Figure 8e). These results indicate that the synergistic ligand system effectively stabilizes [PbBr_6_]^2−^ octahedra on the NC surface and controls the charge recombination pathways. As a result, the MAPbBr_3_ NCs synthesized via this strategy exhibited a PLQY exceeding 99%, and the corresponding LEDs achieved excellent performance with an EQE of 7.8%, current efficiency of 23.7 cd A^−1^, and luminance of 1.59 × 10^4^ cd m^−2^. This study demonstrated that a combination of conjugated and synergistic ligands could simultaneously enhance the structural stability and optoelectronic properties of PeNCs. [80]

Li et al. introduced a conjugated diphenylphosphoryl azide (DPPA) into CsPb(Br/I)_3_ NCs during synthesis to regulate the surface ligand environment, aiming to realize highly luminescent, efficient, and thermally stable pure red PeLEDs. DPPA contains a π-conjugated benzene ring with excellent electrical conductivity and a phosphonate radical with a high binding affinity to the NC surface, thereby facilitating charge transport and surface passivation [81]. During synthesis, DPPA reacts with carboxylic acids to generate diphenyl phosphate, which acts as a substitute ligand bound to the NC surface (Figure 8f). Additionally, its reaction with OLA enabled the anchoring of aromatic ligands on the NC surface while simultaneously consuming long-chain carboxylic acids and OLA. This process reduces the concentration of –RNH_3_^+^ species in the precursor solution, which suppresses surface termination during crystallization and promotes the Cs^+^-dominated NC growth. As a result, the DPPA-treated NCs exhibited a larger average particle size than that of pristine NCs, which was favorable for charge carrier transport. This study demonstrated that DPPA-mediated surface ligand engineering could significantly improve the optoelectronic properties and thermal stability of PeNCs.

The DPPA-treated CsPb(Br/I)_3_ NCs exhibited a red shift of the PL peak from 630 to 638 nm (Figure 8g), which is consistent with an increase in the NC size. TRPL measurements revealed that DPPA NCs displayed a mono-exponential decay with an extended lifetime of 20.9 ns, whereas pristine NCs showed a shorter lifetime of 11.8 ns and significantly higher nonradiative recombination rate (Figure 8h).

These improved surface properties translated into better device performance. J–V–L characteristics revealed that the DPPA-NC-based PeLEDs exhibited a lower turn-on voltage and higher luminance across the entire operational range, indicating improved charge transport and superior optoelectronic properties. As a result, the devices achieved a maximum EQE of 24.8% (Figure 8i,j), demonstrating significant efficiency and operational stability improvements as compared with those of the pristine devices.

#### 2.3.2. X-Type Ligands

X-type ligands generally possess anionic characteristics and donate single electron to coordinate with central metal ions. In PeNCs, X-type ligands form bonds by sharing single electrons with NC surface atoms. Compared with the coordination bonds formed by L-type or Z-type ligands, these chemical bonds are typically more stable, thereby strengthening the ligand–NC interaction and effectively contributing to stability enhancement. Consequently, X-type ligands are advantageous for passivating halide vacancies, reducing nonradiative recombination, and improving the device performance. Representative X-type ligands include carboxylates (–COO^−^), thiolates (S^−^), halides (Cl^−^, Br^−^), and sulfonates (–SO_3_^−^) [82,83].

**Figure 9:**
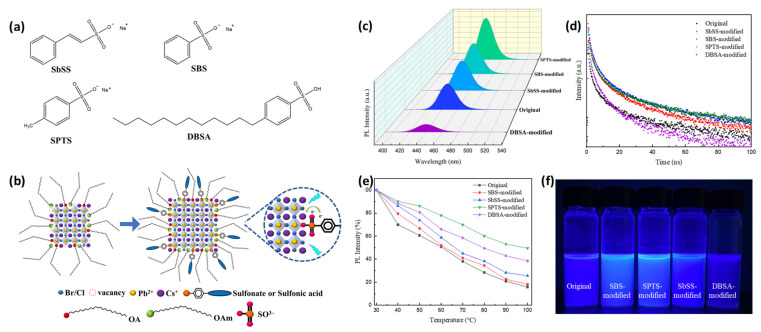
(**a**,**b**) Chemical structures of sodium β-styrenesulfonate (SbSS), sodium benzenesulfonate (SBS), sodium p-toluenesulfonate (SPTS), and 4-dodecylbenzenesulfonic acid (DBSA). (**c**) PL emission spectra, (**d**) TRPL decay curves, (**e**) temperature-dependent PL intensity evolution, and (**f**) solutions of the PeNCs exposed to UV light (375 nm). Reprinted with permission from Ref. [84] © 1996–2025 MDPI (Basel, Switzerland) unless stated otherwise.

Huang et al. proposed a surface passivation strategy for blue-emitting CsPbBr_1.5_Cl_1.5_ NCs by introducing sulfonic acid or sulfonate ligands into a Pb precursor solution (Figure 9a). Blue emission from PeNCs can be achieved by reducing the particle size. However, as the particle size decreases, the surface-to-volume ratio sharply increases, resulting in a higher number of surface defects and, consequently, a lower PLQY. In addition, blue PL emission in mixed-halide PeNCs can be tuned by adjusting the Cl^−^ ion content. Nevertheless, increasing the Cl content tends to induce the formation of deep trap states within the bandgap owing to the presence of Cl-related defects, which reduce the spectral stability. In this study, sulfonate-containing ligands bearing S=O functional groups, such as sodium β-styrenesulfonate (SbSS), sodium benzenesulfonate (SBS), sodium p-toluenesulfonate (SPTS), and 4-dodecylbenzenesulfonic acid (DBSA), were employed to effectively passivate halide vacancies (Figure 9b). This strategy increased the PL intensity and stability [84].

According to the PL spectra (Figure 9c), blue emission was retained even after the incorporation of SbSS, SBS, SPTS, and DBSA. Moreover, the PL intensities of the NCs treated with SbSS, SBS, and SPTS were enhanced as compared with those of the original NCs. The PLQY increased from 32% for the untreated NCs to 48%, 38%, and 63% after the treatments with SbSS, SBS, and SPTS, respectively. This improvement was attributed to a reduction in the number of surface defects and enhanced radiative recombination following sulfonate ligand incorporation. However, the DBSA-treated NCs exhibited the lowest PL intensity (Figure 9c,f), while the PLQY value decreased to 18%. This performance degradation is attributed to the reduced crystal size caused by DBSA, which increases the surface area and, consequently, the density of surface traps. The results of PL and TRPL measurements revealed that the NCs treated with SPTS, SBS, and SbSS ligands exhibited longer charge-carrier lifetimes (Figure 9d). During thermal stability evaluation, the SPTS-modified NCs retained approximately 80% of their initial PL intensity even at 60 °C, surpassing the performance of the untreated NCs (Figure 9e) [84].

Thiol ligands are considered effective passivating agents for enhancing optoelectronic properties because of their strong affinity for coordinated Pb atoms. Uddin et al. reported that introducing 4-hydroxy-6-(trifluoromethyl)pyrimidine-2-thiol (P-CF_3_–OH–SH), a molecule bearing both hydroxyl (–OH) and trifluoromethyl (–CF_3_) groups, resulted in the most significant improvement in the PL intensity, stability, and photovoltaic performance of Cs_0.15_FA_0.85_PbI_3_ PeNCs. In this work, pyrimidine-based ligands were designed and introduced into PeNCs. The presence of thiol groups (–SH) significantly increased the PL intensity, and the incorporation of the electron-withdrawing trifluoromethyl (CF_3_) group further amplified this enhancement by approximately seven times. XPS and NMR spectra revealed that pyrimidine molecules containing thiol groups underwent deprotonation in solution to form thiolates (SH → S^−^), thereby strengthening the passivation effect [85].

#### 2.3.3. Z-Type Ligands

Unlike L-type and X-type ligands that donate electrons, Z-type ligands accept two electrons. They typically bind to halide anions on the surface of PeNCs, thereby passivating surface defects. Well-known Z-type ligands include K^+^, Na^+^, Mg^2+^, and Zn^2+^ ions. Although these species are cationic in nature, they are defined as Z-type ligands when they are not introduced into the perovskite lattice as dopants at A-sites or B-sites, but instead remain on the NC surface and form coordination bonds by accepting electrons from surface halides [86]. Metal ion doping in perovskite structures generally increases the tolerance factor, thereby facilitating the formation of a stable [PbI_6_]^4−^ octahedral framework. This approach has been established as an effective strategy for enhancing the PL stability of CsPbX_3_ NCs [87]. However, excessive doping can disrupt the distribution and coordination of lattice atoms, inducing new defect states that degrade the optical properties and alter the crystal morphology [88]. Some researchers have reported that metal halide salts can be used to passivate surface defects through their adsorption on the NC surface rather than lattice incorporation.

Cao et al. introduced Mg^2+^ ions onto the surface of CsPbI_3_ NCs to achieve high PL efficiency and enhanced stability. Owing to their significantly smaller ionic radius as compared with that of Pb^2+^, Mg^2+^ ions cannot easily substitute Pb^2+^ ions within the lattice. Therefore, Mg^2+^ is considered a promising agent for the passivation of defects primarily localized on the surface rather than within the crystal lattice [89]. TEM analysis results confirmed that the lattice spacing remained unchanged, indicating that Mg^2+^ ions did not substitute Pb^2+^ ions in the crystal lattice but were predominantly located on the surface. By incorporating a Z-type Mg^2+^ ligand, nonradiative recombination was suppressed, and the PLQY value increased to 95%. Furthermore, NCs retained 80% of their initial PLQY even after 80 d of air exposure, and the red emission along with the cubic-phase structure was maintained for 9 d under ambient conditions in the film state. When employed as the EML in red PeLEDs, Mg–CsPbI_3_ NCs exhibited a maximum EQE of 8.4%, outperforming the devices based on the undoped CsPbI_3_ NCs. This study demonstrated that the Mg^2+^-assisted surface defect passivation strategy helped realize CsPbI_3_ NCs with high PL efficiency and environmental stability [90].

Generally, mixed-halide compositions or low-dimensional MHPs are employed to realize blue emissive MHPs. However, mixed-halide MHPs are prone to severe bias-induced phase segregation in LEDs due to their soft ionic lattice and high halide-ion mobility [91]. In contrast, bromide-based low-dimensional PeLEDs do not exhibit these issues and can achieve deep-blue emission. Nevertheless, quasi-two-dimensional structures also face limitations in achieving pure deep-blue emission owing to the increased nonradiative recombination and electroluminescence color shift. Zero-dimensional PeNCs possess high PLQY and color purity; however, their large surface-to-volume ratio necessitates dense surface ligand passivation. This can impede charge transport, which necessitates the use of relatively short and sparsely packed ligands.

To address these challenges, Chen et al. introduced zinc octanoate as a Z-type ligand to achieve deep blue emission. XPS analysis results confirmed that Zn^2+^ ions were chemically adsorbed on the CsPbBr_3_ surface, while X-ray diffraction patterns demonstrated no peak shifts as compared with those of pristine CsPbBr_3_, indicating no Zn^2+^ incorporation into the crystal lattice. Furthermore, Fourier-transform infrared spectroscopy data revealed redshifts in the C=O and COO^−^ stretching vibrations, which are attributed to the interactions between the surface uncoordinated Br^−^ ions and empty 2p orbitals of Zn^2+^. Consequently, a high PLQY of 73% was achieved in this study, which was attributed to the strong interactions between Zn^2+^ species and both surface Br^−^ ions and carboxyl groups, regulating the acid–base equilibrium and effectively passivating surface defects. This strategy suppresses the overaccumulation of amine ligands on the PeNC surface, inhibits abnormal Br^−^ losses, and enables precise control over the size and morphology of PeNCs in polar solvents [92].

## 3. Conclusions

PeNCs have emerged as promising materials for next-generation light-emitting devices owing to their outstanding PL properties and solution processability at low temperatures. However, their practical applications are limited by the intrinsic and extrinsic instabilities that lead to performance degradation during operation. This review has outlined the primary instability factors—the compositional, thermal, and environmental ones—and discussed how each factor affected the structural and optical properties of PeNCs.

To overcome these limitations, this review highlights three core engineering strategies: compositional engineering, ligand engineering, and surface passivation. First, compositional engineering of A- and B-site ions enhances phase stability and suppresses ion migration, thereby mitigating hysteresis, spectral drift, and bias-related instabilities in PeLEDs, and contributing to stable emission spectra under prolonged device operation [93]. Second, the introduction of X-, L-, and Z-type ligands and NC ligand modification have been reported to improve operational stabilities such as PL property and EQE of devices. In addition, employing functional ligands can enhance charge transport and thin-film robustness, enabling high-resolution patterning and suppression of coffee-ring effects in practical fabrication processing [94]. Third, surface passivation chemically seals surface defects to effectively suppress nonradiative recombination, thereby increasing the PLQY and EQE of PeLEDs and extending operational lifetimes, which in turn enables the development of high-performance, high-efficiency PeNC-based optoelectronic devices.

Future works should focus on synergistically integrating these approaches, developing in situ diagnostic tools, and exploring lead-free alternatives to support environmentally friendly applications. This review provides a framework for the rational design of stable high-performance PeNCs for advanced optoelectronic technologies.

## Figures and Tables

**Table 1:** PL properties and synthesis method of PeNCs.

Materials	PL Peak (nm)	Synthesis Method	PLQY (%)	Reference
CsPbCl_3_	408	Hot injection	65	[11]
CsPbBr_3_	512	Hot injection	92	[11]
CsPbI_3_	691	Hot injection	58	[11]
Cs_4_PbBr_6_	375	two-step dissolution–recrystallization mechanism	-	[15]
FAPbCl_3_	415	LARP	1	[12]
FAPbCl_1.5_Br_1.5_	455	LARP	26	[12]
FAPbBr_3_	526	LARP	84	[12]
FAPbBr_1.5_I_1.5_	630	LARP	21	[12]
FAPbI_3_	740	LARP	55	[12]
MAPbCl_3_	400	Wet grinding	5	[13]
MAPbBr_3_	522	Wet grinding	75	[13]
MAPbI_3_	780	Wet grinding	55	[13]
CsCu_2_I_3_	561	Hot injection	11	[16]
Cs_3_Cu_2_I_5_	440	Hot injection	96.6	[17]
K_3_SbCl_6_	440	Hot injection	22.3	[18]
Cs_2_AgBiBr_6_	625	Hot injection, LARP	-	[19]
FA_3_Bi_2_Br_9_	437	LARP	52	[20]

## Data Availability

No new data were created or analyzed in this study. Data sharing is not applicable to this article.

## References

[1] Yang W.S., Noh J.H., Jeon N.J., Kim Y.C., Ryu S., Seo J., Seok S.I. (2015). High-Performance Photovoltaic Perovskite Layers Fabricated through Intramolecular Exchange. Science.

[2] Dong H., Ran C., Gao W., Li M., Xia Y., Huang W. (2023). Metal Halide Perovskite for Next-Generation Optoelectronics: Progresses and Prospects. eLight.

[3] Zhao J., Ji G., Li Y., Hu R., Zheng J. (2021). Preparation of a Self-Healing Polyaniline-Based Gel and Its Application as a Healable All-in-One Capacitor. Chem. Eng. J..

[4] Feng S., Povilus B., Yang S. (2023). Color Tuning Halide Perovskites: Optical Amplification and Lasing. Mater. Today Adv..

[5] Triana M.A., Hsiang E.L., Zhang C., Dong Y., Wu S.T. (2022). Luminescent Nanomaterials for Energy-Efficient Display and Healthcare. ACS Energy Lett..

[6] Chu S., Chen W., Fang Z., Xiao X., Liu Y., Chen J., Huang J., Xiao Z. (2021). Large-Area and Efficient Perovskite Light-Emitting Diodes via Low-Temperature Blade-Coating. Nat. Commun..

[7] Chouhan L., Ghimire S., Subrahmanyam C., Miyasaka T., Biju V. (2020). Synthesis, Optoelectronic Properties and Applications of Halide Perovskites. Chem. Soc. Rev..

[8] Savill K.J., Ulatowski A.M., Herz L.M. (2021). Optoelectronic Properties of Tin-Lead Halide Perovskites. ACS Energy Lett..

[9] Hoye R.L.Z., Hidalgo J., Jagt R.A., Correa-Baena J.P., Fix T., MacManus-Driscoll J.L. (2022). The Role of Dimensionality on the Optoelectronic Properties of Oxide and Halide Perovskites, and Their Halide Derivatives. Adv. Energy Mater..

[10] Huang H., Bodnarchuk M.I., Kershaw S.V., Kovalenko M.V., Rogach A.L. (2017). Lead Halide Perovskite Nanocrystals in the Research Spotlight: Stability and Defect Tolerance. ACS Energy Lett..

[11] Imran M., Caligiuri V., Wang M., Goldoni L., Prato M., Krahne R., De Trizio L., Manna L. (2018). Benzoyl Halides as Alternative Precursors for the Colloidal Synthesis of Lead-Based Halide Perovskite Nanocrystals. J. Am. Chem. Soc..

[12] Levchuk I., Osvet A., Tang X., Brandl M., Perea J.D., Hoegl F., Matt G.J., Hock R., Batentschuk M., Brabec C.J. (2017). Brightly Luminescent and Color-Tunable Formamidinium Lead Halide Perovskite FAPbX_3_ (X = Cl, Br, I) Colloidal Nanocrystals. Nano Lett..

[13] Chen D., Li J., Chen X., Chen J., Zhong J. (2019). Grinding Synthesis of APbX_3_ (A = MA, FA, Cs; X = Cl, Br, I) Perovskite Nanocrystals. ACS Appl. Mater. Interfaces.

[14] Wei J.H., Wang X.D., Liao J.F., Kuang D. (2020). Bin High Photoluminescence Quantum Yield (>95%) of MAPbBr_3_ Nanocrystals via Reprecipitation from Methylamine-MAPbBr_3_ Liquid. ACS Appl. Electron. Mater..

[15] Liu Z., Bekenstein Y., Ye X., Nguyen S.C., Swabeck J., Zhang D., Lee S.T., Yang P., Ma W., Alivisatos A.P. (2017). Ligand Mediated Transformation of Cesium Lead Bromide Perovskite Nanocrystals to Lead Depleted Cs_4_PbBr_6_ Nanocrystals. J. Am. Chem. Soc..

[16] Qu J., Xu S., Shao H., Xia P., Lu C., Wang C., Ban D. (2023). Recent Progress of Copper Halide Perovskites: Properties, Synthesis and Applications. J. Mater. Chem. C.

[17] Gao F., Zhu X., Feng Q., Zhong W., Liu W., Xu H., Liu Y. (2022). Deep-Blue Emissive Cs_3_Cu_2_I_5_ Perovskites Nanocrystals with 96.6% Quantum Yield via InI_3_-Assisted Synthesis for Light-Emitting Device and Fluorescent Ink Applications. Nano Energy.

[18] Wu Y., Xiang G., Zhang M., Wei D., Cheng C., Leng J., Ma H. (2022). Electronic Structures and Photoelectric Properties in Cs_3_Sb_2_X_9_ (X = Cl, Br, or I) under High Pressure: A First Principles Study. Nanomaterials.

[19] Cai T., Shi W., Hwang S., Kobbekaduwa K., Nagaoka Y., Yang H., Hills-Kimball K., Zhu H., Wang J., Wang Z. (2020). Lead-Free Cs_4_CuSb_2_Cl_12_ Layered Double Perovskite Nanocrystals. J. Am. Chem. Soc..

[20] Shen Y., Yin J., Cai B., Wang Z., Dong Y., Xu X., Zeng H. (2020). Lead-Free, Stable, High-Efficiency (52%) Blue Luminescent FA_3_Bi_2_Br_9_ Perovskite Quantum Dots. Nanoscale Horiz..

[21] Xing K., Cao S., Yuan X., Zeng R., Li H., Zou B., Zhao J. (2021). Thermal and Photo Stability of All Inorganic Lead Halide Perovskite Nanocrystals. Phys. Chem. Chem. Phys..

[22] Almutlaq J., Yin J., Mohammed O.F., Bakr O.M. (2018). The Benefit and Challenges of Zero-Dimensional Perovskites. J. Phys. Chem. Lett..

[23] Zhang H., Fu X., Tang Y., Wang H., Zhang C., Yu W.W., Wang X., Zhang Y., Xiao M. (2019). Phase Segregation Due to Ion Migration in All-Inorganic Mixed-Halide Perovskite Nanocrystals. Nat. Commun..

[24] Ghimire S., Klinke C. (2021). Two-Dimensional Halide Perovskites: Synthesis, Optoelectronic Properties, Stability, and Applications. Nanoscale.

[25] Li X., Cao F., Yu D., Chen J., Sun Z., Shen Y., Zhu Y., Wang L., Wei Y., Wu Y. (2017). All Inorganic Halide Perovskites Nanosystem: Synthesis, Structural Features, Optical Properties and Optoelectronic Applications. Small.

[26] Li X., Gao X., Zhang X., Shen X., Lu M., Wu J., Shi Z., Colvin V.L., Hu J., Bai X. (2021). Lead-Free Halide Perovskites for Light Emission: Recent Advances and Perspectives. Adv. Sci..

[27] Dutt V.G.V., Akhil S., Mishra N. (2020). Surface Passivation Strategies for Improving Photoluminescence and Stability of Cesium Lead Halide Perovskite Nanocrystals. ChemNanoMat.

[28] Shao Y., Fang Y., Li T., Wang Q., Dong Q., Deng Y., Yuan Y., Wei H., Wang M., Gruverman A. (2016). Grain Boundary Dominated Ion Migration in Polycrystalline Organic-Inorganic Halide Perovskite Films. Energy Environ. Sci..

[29] Kamat P.V., Kuno M. (2021). Halide Ion Migration in Perovskite Nanocrystals and Nanostructures. Acc. Chem. Res..

[30] Han T.H., Tan S., Xue J., Meng L., Lee J.W., Yang Y. (2019). Interface and Defect Engineering for Metal Halide Perovskite Optoelectronic Devices. Adv. Mater..

[31] Aftabuzzaman M., Hong Y., Jeong S., Levan R., Lee S.J., Choi D.H., Lee K. (2025). Colloidal Perovskite Nanocrystals for Blue-Light-Emitting Diodes and Displays. Adv. Sci..

[32] Wang Y., Ren J., Zhou X., Zhang G. (2023). Stability Improvements of Metal Halide Perovskite Nanocrystals and Their Optoelectrical Applications. Mater. Chem. Front..

[33] Aristidou N., Sanchez-Molina I., Chotchuangchutchaval T., Brown M., Martinez L., Rath T., Haque S.A. (2015). The Role of Oxygen in the Degradation of Methylammonium Lead Trihalide Perovskite Photoactive Layers. Angew. Chem..

[34] Konidakis I., Karagiannaki A., Stratakis E. (2022). Advanced Composite Glasses with Metallic, Perovskite, and Two-Dimensional Nanocrystals for Optoelectronic and Photonic Applications. Nanoscale.

[35] Wang S., Yousefi Amin A.A., Wu L., Cao M., Zhang Q., Ameri T. (2021). Perovskite Nanocrystals: Synthesis, Stability, and Optoelectronic Applications. Small Struct..

[36] Otero-Martínez C., Fiuza-Maneiro N., Polavarapu L. (2022). Enhancing the Intrinsic and Extrinsic Stability of Halide Perovskite Nanocrystals for Efficient and Durable Optoelectronics. ACS Appl. Mater. Interfaces.

[37] Becker M., Klüner T., Wark M. (2017). Formation of Hybrid ABX_3_ Perovskite Compounds for Solar Cell Application: First-Principles Calculations of Effective Ionic Radii and Determination of Tolerance Factors. Dalt. Trans..

[38] Travis W., Glover E.N.K., Bronstein H., Scanlon D.O., Palgrave R.G. (2016). On the Application of the Tolerance Factor to Inorganic and Hybrid Halide Perovskites: A Revised System. Chem. Sci..

[39] Burger S., Ehrenreich M.G., Kieslich G. (2018). Tolerance Factors of Hybrid Organic-Inorganic Perovskites: Recent Improvements and Current State of Research. J. Mater. Chem. A.

[40] Huang W., Zhou Z., Nam S.H., Chen Q., Wang J., Zeng Z., Ge C., Li Y., Wang J., Kim Y.H. (2025). Spin Lifetime in Hybrid Organic-Inorganic Perovskites: Mechanisms, Measurements, and Prospects for Spintronic Applications. J. Phys. Chem. Lett..

[41] Livakas N., Toso S., Ivanov Y.P., Das T., Chakraborty S., Divitini G., Manna L. (2023). CsPbCl_3_ → CsPbI_3_ Exchange in Perovskite Nanocrystals Proceeds through a Jump-the-Gap Reaction Mechanism. J. Am. Chem. Soc..

[42] Van der Stam W., Geuchies J.J., Altantzis T., Van Den Bos K.H.W., Meeldijk J.D., Van Aert S., Bals S., Vanmaekelbergh D., De Mello Donega C. (2017). Highly Emissive Divalent-Ion-Doped Colloidal CsPb_1-X_M_x_Br_3_ Perovskite Nanocrystals through Cation Exchange. J. Am. Chem. Soc..

[43] Amat A., Mosconi E., Ronca E., Quarti C., Umari P., Nazeeruddin M.K., Grätzel M., De Angelis F. (2014). Cation-Induced Band-Gap Tuning in Organohalide Perovskites: Interplay of Spin-Orbit Coupling and Octahedra Tilting. Nano Lett..

[44] Yang B., Bogachuk D., Suo J., Wagner L., Kim H., Lim J., Hinsch A., Boschloo G., Nazeeruddin M.K., Hagfeldt A. (2022). Strain Effects on Halide Perovskite Solar Cells. Chem. Soc. Rev..

[45] Protesescu L., Yakunin S., Kumar S., Bär J., Bertolotti F., Masciocchi N., Guagliardi A., Grotevent M., Shorubalko I., Bodnarchuk M.I. (2017). Dismantling the “Red Wall” of Colloidal Perovskites: Highly Luminescent Formamidinium and Formamidinium-Cesium Lead Iodide Nanocrystals. ACS Nano.

[46] Serafini P., Villanueva-Antolí A., Adhikari S.D., Masi S., Sánchez R.S., Rodriguez-Pereira J., Pradhan B., Hofkens J., Gualdrón-Reyes A.F., Mora-Seró I. (2023). Increasing the Performance and Stability of Red-Light-Emitting Diodes Using Guanidinium Mixed-Cation Perovskite Nanocrystals. Chem. Mater..

[47] Vashishtha P., Veldhuis S.A., Dintakurti S.S.H., Kelly N.L., Griffith B.E., Brown A.A.M., Ansari M.S., Bruno A., Mathews N., Fang Y. (2020). Investigating the Structure-Function Relationship in Triple Cation Perovskite Nanocrystals for Light-Emitting Diode Applications. J. Mater. Chem. C.

[48] Saliba M., Matsui T., Seo J.Y., Domanski K., Correa-Baena J.P., Nazeeruddin M.K., Zakeeruddin S.M., Tress W., Abate A., Hagfeldt A. (2016). Cesium-Containing Triple Cation Perovskite Solar Cells: Improved Stability, Reproducibility and High Efficiency. Energy Environ. Sci..

[49] Ko P.K., Chen D., Li C.H.A., Chan C.C.S., Sergeev A., Ding P., Lam D., Ouyang B., Guo L., Wong K.S. (2024). Color Tuning in Cesium Lead Halide Nanocrystals via A-Site Substitution as an Alternative Method for Achieving Spectrally Stable Blue Perovskite Nanocrystal Light-Emitting Diodes. Chem. Mater..

[50] Lu Y., Alam F., Shamsi J., Abdi-Jalebi M. (2024). Doping Up the Light: A Review of A/B-Site Doping in Metal Halide Perovskite Nanocrystals for Next-Generation LEDs. J. Phys. Chem. C.

[51] Luo B., Li F., Xu K., Guo Y., Liu Y., Xia Z., Zhang J.Z. (2019). B-Site Doped Lead Halide Perovskites: Synthesis, Band Engineering, Photophysics, and Light Emission Applications. J. Mater. Chem. C.

[52] Zeng Y.-T., Li Z.-R., Chang S.-P., Ansay A., Wang Z.-H., Huang C.-Y. (2022). Bright CsPbBr_3_ Perovskite Nanocrystals with Improved Stability by In-Situ Zn-Doping. Nanomaterials.

[53] Das S., De A., Samanta A. (2020). Ambient Condition Mg^2+^ Doping Producing Highly Luminescent Green-and Violet-Emitting Perovskite Nanocrystals with Reduced Toxicity and Enhanced Stability. J. Phys. Chem. Lett..

[54] Kim H., Bae S.R., Lee T.H., Lee H., Kang H., Park S., Jang H.W., Kim S.Y. (2021). Enhanced Optical Properties and Stability of CsPbBr_3_ Nanocrystals Through Nickel Doping. Adv. Funct. Mater..

[55] Chen Z., Zhou B., Yuan J., Tang N., Lian L., Qin L., Zhu L., Zhang J., Chen R., Zang J. (2021). Cu^2+^-Doped CsPbI_3_ Nanocrystals with Enhanced Stability for Light-Emitting Diodes. J. Phys. Chem. Lett..

[56] Guvenc C.M., Yalcinkaya Y., Ozen S., Sahin H., Demir M.M. (2019). Gd^3+^-Doped α-CsPbI_3_ Nanocrystals with Better Phase Stability and Optical Properties. J. Phys. Chem. C.

[57] Ten Brinck S., Zaccaria F., Infante I. (2019). Defects in Lead Halide Perovskite Nanocrystals: Analogies and (Many) Differences with the Bulk. ACS Energy Lett..

[58] Li X., Cai W., Guan H., Zhao S., Cao S., Chen C., Liu M., Zang Z. (2021). Highly Stable CsPbBr_3_ Quantum Dots by Silica-Coating and Ligand Modification for White Light-Emitting Diodes and Visible Light Communication. Chem. Eng. J..

[59] Cao W., Liu P., Chen W., Wang W., Xu B., Wu D., Hao J., Cao W., Fang F., Li Y. (2017). Halide-Rich Synthesized Cesium Lead Bromide Perovskite Nanocrystals for Light-Emitting Diodes with Improved Performance. Chem. Mater..

[60] Ahmed G.H., El-Demellawi J.K., Yin J., Pan J., Velusamy D.B., Hedhili M.N., Alarousu E., Bakr O.M., Alshareef H.N., Mohammed O.F. (2018). Giant Photoluminescence Enhancement in CsPbCl_3_ Perovskite Nanocrystals by Simultaneous Dual-Surface Passivation. ACS Energy Lett..

[61] Yang D., Li X., Zeng H. (2018). Surface Chemistry of All Inorganic Halide Perovskite Nanocrystals: Passivation Mechanism and Stability. Adv. Mater. Interfaces.

[62] Shamsi J., Urban A.S., Imran M., De Trizio L., Manna L. (2019). Metal Halide Perovskite Nanocrystals: Synthesis, Post-Synthesis Modifications, and Their Optical Properties. Chem. Rev..

[63] Saleem M.I., Katware A., Amin A., Jung S.H., Lee J.H. (2023). YCl_3_-Substituted CsPbI_3_ Perovskite Nanorods for Efficient Red-Light-Emitting Diodes. Nanomaterials.

[64] Ahmed T., Seth S., Samanta A. (2018). Boosting the Photoluminescence of CsPbX_3_ (X = Cl, Br, I) Perovskite Nanocrystals Covering a Wide Wavelength Range by Postsynthetic Treatment with Tetrafluoroborate Salts. Chem. Mater..

[65] Zheng K., Žídek K., Abdellah M., Messing M.E., Al-Marri M.J., Pullerits T. (2016). Trap States and Their Dynamics in Organometal Halide Perovskite Nanoparticles and Bulk Crystals. J. Phys. Chem. C.

[66] Huang H., Raith J., Kershaw S.V., Kalytchuk S., Tomanec O., Jing L., Susha A.S., Zboril R., Rogach A.L. (2017). Growth Mechanism of Strongly Emitting CH_3_NH_3_PbBr_3_ Perovskite Nanocrystals with a Tunable Bandgap. Nat. Commun..

[67] Tan Y., Zou Y., Wu L., Huang Q., Yang D., Chen M., Ban M., Wu C., Wu T., Bai S. (2018). Highly Luminescent and Stable Perovskite Nanocrystals with Octylphosphonic Acid as a Ligand for Efficient Light-Emitting Diodes. ACS Appl. Mater. Interfaces.

[68] De Roo J., Ibáñez M., Geiregat P., Nedelcu G., Walravens W., Maes J., Martins J.C., Van Driessche I., Kovalenko M.V., Hens Z. (2016). Highly Dynamic Ligand Binding and Light Absorption Coefficient of Cesium Lead Bromide Perovskite Nanocrystals. ACS Nano.

[69] Ravi V.K., Santra P.K., Joshi N., Chugh J., Singh S.K., Rensmo H., Ghosh P., Nag A. (2017). Origin of the Substitution Mechanism for the Binding of Organic Ligands on the Surface of CsPbBr_3_ Perovskite Nanocubes. J. Phys. Chem. Lett..

[70] Wu L., Zhong Q., Yang D., Chen M., Hu H., Pan Q., Liu H., Cao M., Xu Y., Sun B. (2017). Improving the Stability and Size Tunability of Cesium Lead Halide Perovskite Nanocrystals Using Trioctylphosphine Oxide as the Capping Ligand. Langmuir.

[71] Liu H., Wu Z., Gao H., Shao J., Zou H., Yao D., Liu Y., Zhang H., Yang B. (2017). One-Step Preparation of Cesium Lead Halide CsPbX_3_ (X = Cl, Br, and I) Perovskite Nanocrystals by Microwave Irradiation. ACS Appl. Mater. Interfaces.

[72] Tang Y., Wu Z., Zhang X., Liao K., Wang G. (2024). Synergistic Passivation Inducing Long-Term Stability for Fluorescent CsPbI_3_ Perovskite Nanocrystals. Adv. Opt. Mater..

[73] Hills-Kimball K., Yang H., Cai T., Wang J., Chen O. (2021). Recent Advances in Ligand Design and Engineering in Lead Halide Perovskite Nanocrystals. Adv. Sci..

[74] Zu Y., Zhang T., Jing L., Gao H., Wang W., Yang K., Zeng B., Gan R., Gong D., Liu P. (2025). Bifunctional Ligand Passivation Enables Robust-Stability Perovskite Nanocrystals for Backlit Display. Dyes Pigment..

[75] Pradhan J., Moitra P., Das B., Mondal P., Kumar G.S., Ghorai U.K., Acharya S., Bhattacharya S. (2020). Encapsulation of CsPbBr_3_ Nanocrystals by a Tripodal Amine Markedly Improves Photoluminescence and Stability Concomitantly via Anion Defect Elimination. Chem. Mater..

[76] Yuan L., Chen D., He K., Xu J., Xu K., Hu J., Liang S., Zhu H. (2024). Advancing Microarray Fabrication: One-Pot Synthesis and High-Resolution Patterning of UV-Crosslinkable Perovskite Quantum Dots. Nano Res..

[77] Oh B.M., Jeong Y., Zheng J., Cho N.Y., Song M., Choi J.W., Kim J.H. (2021). Simple One-Pot Synthesis and High-Resolution Patterning of Perovskite Quantum Dots Using a Photocurable Ligand. Chem. Commun..

[78] Wächtler E., Gericke R., Brendler E., Gerke B., Langer T., Pöttgen R., Zhechkov L., Heine T., Wagler J. (2016). Group 10-Group 14 Metal Complexes [E-TM]IV: The Role of the Group 14 Site as an L, X and Z-Type Ligand. Dalt. Trans..

[79] Sun W., Yun R., Liu Y., Zhang X., Yuan M., Zhang L., Li X. (2023). Ligands in Lead Halide Perovskite Nanocrystals: From Synthesis to Optoelectronic Applications. Small.

[80] Zhao C., Dai J., Zhu C., Liu X., Dong H., Yuan F., Jiao B., Yu Y., Wu Z. (2022). Complementary Triple-Ligand Engineering Approach to Methylamine Lead Bromide Nanocrystals for High-Performance Light-Emitting Diodes. ACS Appl. Mater. Interfaces.

[81] Li H., Zhu X., Zhang D., Gao Y., Feng Y., Ma Z., Huang J., He H., Ye Z., Dai X. (2024). Thermal Management towards Ultra-Bright and Stable Perovskite Nanocrystal-Based Pure Red Light-Emitting Diodes. Nat. Commun..

[82] Ruan L., Shen W., Wang A., Zhou Q., Zhang H., Deng Z. (2017). Stable and Conductive Lead Halide Perovskites Facilitated by X-Type Ligands. Nanoscale.

[83] Kuan C.H., Yang S.H. (2022). Surface Ligand Engineering of Perovskite Nanocrystals with a Conjugated Sulfonate Ligand for Light-Emitting Applications. Mater. Adv..

[84] Huang S.H., Yang S.H., Tsai W.C., Hsu H.C. (2024). Enhancing Optical and Thermal Stability of Blue-Emitting Perovskite Nanocrystals through Surface Passivation with Sulfonate or Sulfonic Acid Ligands. Nanomaterials.

[85] Uddin M.A., Hossain T., Kothalawala N.L., Joy S., Kim D.Y., Graham K.R. (2022). Multifunctional Thiol-Containing Additives for Improved Photoluminescence and Photovoltaic Performance of Cs_0.15_FA_0.85_PbI_3_ Perovskites. ACS Appl. Electron. Mater..

[86] Fiuza-Maneiro N., Sun K., López-Fernández I., Gómez-Graña S., Müller-Buschbaum P., Polavarapu L. (2023). Ligand Chemistry of Inorganic Lead Halide Perovskite Nanocrystals. ACS Energy Lett..

[87] Zhang N., Xia K., He Q., Pan J. (2022). Recent Progress in the Stability of Red-Emissive Perovskite Nanocrystals for Light-Emitting Diodes. ACS Mater. Lett..

[88] Behera R.K., Dutta A., Ghosh D., Bera S., Bhattacharyya S., Pradhan N. (2019). Doping the Smallest Shannon Radii Transition Metal Ion Ni(II) for Stabilizing α-CsPbI3 Perovskite Nanocrystals. J. Phys. Chem. Lett..

[89] Navas J., Sánchez-Coronilla A., Gallardo J.J., Cruz Hernández N., Piñero J.C., Alcántara R., Fernández-Lorenzo C., De Los Santos D.M., Aguilar T., Martín-Calleja J. (2015). New Insights into Organic-Inorganic Hybrid Perovskite CH_3_NH_3_PbI_3_ Nanoparticles. An Experimental and Theoretical Study of Doping in Pb^2+^ Sites with Sn^2+^, Sr^2+^, Cd^2+^ and Ca^2+^. Nanoscale.

[90] Chen Q., Cao S., Xing K., Ning M., Zeng R., Wang Y., Zhao J. (2021). Mg^2+^-Assisted Passivation of Defects in CsPbI3Perovskite Nanocrystals for High-Efficiency Photoluminescence. J. Phys. Chem. Lett..

[91] Choe H., Jeon D., Lee S.J., Cho J. (2021). Mixed or Segregated: Toward Efficient and Stable Mixed Halide Perovskite-Based Devices. ACS Omega.

[92] Chen Y., Nan M., He Y., Lu S., Shen W., Cheng G., Chen S., Huang W. (2024). Z-Type Ligand Enables Efficient and Stable Deep-Blue Perovskite Light-Emitting Diodes. ACS Appl. Mater. Interfaces.

[93] Sakhatskyi K., John R.A., Guerrero A., Tsarev S., Sabisch S., Das T., Matt G.J., Yakunin S., Cherniukh I., Kotyrba M. (2022). Assessing the Drawbacks and Benefits of Ion Migration in Lead Halide Perovskites. ACS Energy Lett..

[94] Chen Z., Man Z., Rao S., Zhao J., Wang S., Zhang R., Teng F., Tang A. (2025). Rigid Crosslinker-Assisted Nondestructive Direct Photolithograph for Patterned QLED Displays. Light Sci. Appl..

